# Age at Weaning and Infant Growth: Primary Analysis and Systematic Review

**DOI:** 10.1016/j.jpeds.2015.05.003

**Published:** 2015-08

**Authors:** Brennan Vail, Philippa Prentice, David B. Dunger, Ieuan A. Hughes, Carlo L. Acerini, Ken K. Ong

**Affiliations:** 1 Department of Public Health and Primary Care, University of Cambridge, Cambridge, United Kingdom; 2 School of Medicine, University of California San Francisco, San Francisco, CA; 3 Department of Pediatrics, University of Cambridge, Cambridge, United Kingdom; 4 Medical Research Council Epidemiology Unit, Institute of Metabolic Science, Cambridge, United Kingdom

**Keywords:** BMI, Body mass index, CBGS, Cambridge Baby Growth Study, WHO, World Health Organization

## Abstract

**Objective:**

To test whether earlier age at weaning (age 3-6 months) may promote faster growth during infancy.

**Study design:**

Weaning at age 3.0-7.0 months was reported by 571 mothers of term singletons in a prospective birth cohort study conducted in Cambridge, UK. Infant weight and length were measured at birth and at age 3 months and 12 months. Anthropometric values were transformed into age- and sex-adjusted *z*-scores. Three linear regression models were performed, including adjustment for confounders in a stepwise manner. Measurements at age 3 months, before weaning, were used to consider reverse causality.

**Results:**

Almost three-quarters (72.9%) of infants were weaned before age 6 months. Age at weaning of 3.0-7.0 months was inversely associated with weight and length (but not with body mass index) at 12 months (both *P* ≤ .01, adjusted for maternal and demographic factors). These associations were attenuated after adjustment for type of milk feeding and weight or length at age 3 months (before weaning). Rapid weight gain between 0 and 3 months predicted subsequent earlier age at weaning (*P* = .01). Our systematic review identified 2 trials, both reporting null effects of age at weaning on growth, and 15 observational studies, with 10 reporting an inverse association between age at weaning and infant growth and 4 reporting evidence of reverse causality.

**Conclusion:**

In high-income countries, weaning between 3 and 6 months appears to have a neutral effect on infant growth. Inverse associations are likely related to reverse causality.

The introduction of semisolid or solid foods to an infant, whether breast-fed or formula milk-fed, is an important dietary transition (termed here “weaning”). Smooth foods are typically introduced first, followed by lumpy and finger foods. It should be noted that age at weaning as defined here is not synonymous with the duration of exclusive breastfeeding, because many infants are also given formula milk before being introduced to complementary foods.

Previously, the World Health Organization (WHO) recommended that infants be exclusively breastfed for at least 4 months and subsequently introduced to complementary foods at age 4-6 months.^1^ In 2001, the WHO updated this guideline to recommend that infants be exclusively breastfed for the first 6 months of life and then introduced to complementary foods.^2-4^ The WHO also currently recommends that formula milk fed infants be introduced to complementary foods beginning at age 6 months.^5^

The appropriateness of the updated WHO recommendation for high-income countries, where concerns about food safety and availability, as well as infectious diseases, are less prevalent, is a matter of current debate.^6^ The American Academy of Pediatrics and the European Society for Pediatric Gastroenterology, Hepatology, and Nutrition both have expressed general support for the updated WHO recommendations; however, both organizations suggest that complementary foods may be introduced at age 4-6 months, depending on the achievement of developmental milestones and the availability of safe complementary foods.^6-8^ The United Kingdom, Australia, and Canada have adopted the updated WHO recommendation,^9-11^ but adherence is poor. In the most recent UK Infant Feeding Survey conducted in 2010, 7 years after adoption of the current WHO recommendation, only 30% of mothers had introduced complementary foods by age 4 months, and 75% had done so by age 5 months.^9^

The present study focuses on one aspect of the multifaceted debate over the optimal age at weaning: the impact on growth during infancy. We report a primary analysis of the association between age at weaning and infant growth, as well as a review of the existing literature. Our primary analysis addressed 3 themes highlighted in the literature. First, we investigated the suggestion that earlier weaning is associated with faster infant growth and weight gain.^12-14^ Second, we explored the potential role of reverse causality in the relationship between early age at weaning and faster infant growth.^15^ Third, we examined the possible interaction between age at weaning and type of preweaning milk feeding (breast or formula) on infant growth.^16,17^ Our primary analysis and literature review focused largely on the comparison of age at weaning between 3 and 6 months. Studies of very early weaning (age ≤3 months) are discussed only briefly.

## Methods

The primary analysis was based on participants from the Cambridge Baby Growth Study (CBGS), a prospective longitudinal birth cohort study. Mothers were recruited from ultrasound clinics at Rosie Maternity Hospital in Cambridge, UK, between August 2001 and August 2009. Mothers aged <16 years and mothers unable to give informed consent were excluded from the study. Mother–infant pairs were included in this analysis if they met the following inclusion criteria: (1) full-term birth (≥36 weeks); (2) singleton birth; (3) recorded age at weaning between 3.0 and 7.0 months; and (4) recorded anthropometric measurements at birth, 3 months, and 12 months. A total of 571 mother–infant pairs met these inclusion criteria out of the 1121 CBGS pairs asked to record age at weaning at age 12 months (from August 2005 onward). The study was approved by the local Cambridge Research Ethics Committee, and all mothers gave written informed consent.

### Anthropometry

Infant weight and length were measured at birth, 3 months, and 12 months by trained pediatric research nurses. Weight was measured with electronic scales to the nearest 1 g. Supine length was measured with a Kiddimeter (Holtain Ltd, Crymych, UK) to the nearest 0.1 cm. Body mass index (BMI) was calculated using the formula weight (kg)/height (m^2^). Following the current national recommendations, weight, length, and BMI at birth were transformed into age and sex-adjusted *z*-scores by comparison with the British 1990 Growth Reference,^18^ and weight, length, and BMI at age 3 months and 12 months were transformed into age- and sex-adjusted *z*-scores by comparison with the 2006 WHO growth standard.^19^

### Dietary Assessment

Type of milk feeding at the infant's 3-month research clinic visit was assessed by questionnaire. Infants were categorized into 3 groups: exclusively breastfed, exclusively formula fed, or mixed fed at 3 months. At 12 months, mothers retrospectively reported when their infant had first been introduced to smooth, lumpy, and finger foods. For each infant, the earliest of these 3 ages was defined as the age at weaning, which was then classified as 3.00-3.99, 4.0-4.9, 5.0-5.9, and 6.0-6.9 months.

### Statistical Analyses

The Pearson χ^2^ test and ANOVA were used to compare maternal and infant characteristics across the 4 age at weaning groups. ANOVA also was used to compare mean weight, length, and BMI z-scores at birth and at age 3 months and 12 months across the age at weaning groups. Multiple linear regression models were applied to assess the linear association between age at weaning and anthropometric *z*-scores at birth and at age 3 months and 12 months. Three models were performed. Model 1 adjusted only for potential demographic confounders: infant age and sex (*z*-scores used) and maternal age, parity, and deprivation score. Maternal smoking status, marital status, education level, and prepregnancy BMI were not included in the model, because the number of mothers in 1 or more categories was insufficient. Model 2 was also adjusted for type of milk feeding at age 3 months. Model 3 was also adjusted for the same growth outcome measurement (weight, length, or BMI), but at the preceding time point (birth or 3 months). To test the interaction between age at weaning and type of milk feeding at 3 months (breast vs formula), the product of these variables was entered as an additional variable into model 2. All statistical analyses were performed using SPSS version 22 (IBM, Armonk, New York).

### Literature Review

A systematic search was carried out in PubMed and Web of Science for the following terms: infant* AND (growth OR length OR height OR weight OR BMI) AND (time OR timing) AND (complementary OR wean*) AND (food* OR feed*).

Articles were reviewed from database inception through June 30, 2014. Additional studies were retrieved via hand searches of publication lists of selected studies and review articles. Studies had to meet the following inclusion criteria: (1) conducted in a high-income country in North America, Europe, or Australia; (2) participants full-term, single births not selected by disease or risk group; (3) exposure defined as age at introduction of solids, not duration of exclusive breastfeeding or introduction of specific solids; (4) anthropometric outcomes measured at or before age 24 months; (5) papers written in English; and (6) nonduplication of reported results. A formal meta-analysis was not possible owing to heterogeneity in the categorization of age at weaning and in the timing of growth outcome measures among included studies. Instead, results were summarized in table format.

## Results

Of the 571 infants included, 44 (7.7%) were weaned at age 3.0-3.9 months, 146 (25.6%) at age 4.0-4.9 months, 226 (39.6%) at age 5.0-5.9 months, and 155 (27.1%) at age 6.0-6.9 months. Earlier age at weaning was associated with male sex, formula milk feeding, and younger maternal age (all *P* < .01; Table I). Similar trends were observed with lower maternal education level and higher prepregnancy BMI, but the numbers in some of these categories were insufficient to meet the assumptions of the Pearson χ^2^ test.

In unadjusted models, mean weight *z*-scores at birth (*P* = .02), 3 months (*P* = .01), and 12 months (*P* = .01) were significantly different among the age at weaning groups (Table II). At all ages, infants weaned earlier had a higher mean weight *z*-score than infants weaned later. Similar trends were seen for BMI at age 3 months (*P* = .02) and for length at age 12 months (*P* < .01).

In the linear regression model adjusted for age, sex, maternal age, parity, and deprivation score (Table III, model 1), age at weaning was inversely associated with weight at 3 months (*P* = .01) and at 12 months (*P* = .01). In addition, age at weaning was inversely associated with BMI at birth (*P* = .02) and at 3 months (*P* = .01) and also with length at 12 months (*P* < .01). These associations were attenuated after additional adjustment for type of milk feeding at 3 months and were further attenuated after additional adjustment for growth measurements at the preceding time point (ie, before age at weaning) (Table III, models 2 and 3).

When infants were stratified according to milk feeding at age 3 months (exclusive breast vs exclusive or partial formula milk), all associations between age at weaning and infant growth showed similar effect sizes, but these did not reach statistical significance (Table IV; available at www.jpeds.com). Furthermore, we found no evidence of interaction between age at weaning and type of milk feeding at age 3 months on infant growth (data not shown).

To formally test the possibility of reverse causality, we categorized infants according to their change in weight *z*-score (<−0.67, −0.67 to +0.67, or >+0.67) between birth and 3 months.^20^ Infants exhibiting faster weight gain were weaned earlier than those with average or slower weight gain (*P*_trend_ = .01, adjusted for age, sex, maternal age, parity, deprivation score, and weight at birth) (Figure; available at www.jpeds.com).

### Literature Review

Our systematic search identified 488 articles in PubMed and 268 articles in Web of Science. After assessment of titles and abstracts, as well as full text where necessary, 9 articles (8 from PubMed and 1 from Web of Science) met the inclusion criteria. Hand searches of publication lists of the selected studies and other review articles identified 10 additional studies. In total, 19 studies were included in the literature review (Table V). Of these 19 studies, 17 examined infant weaning between age 3 and 6+ months, and the other 2 looked only at very early weaning (≤3 months vs >3 months).

Two randomized controlled trials investigated the effect of age at weaning on infant growth.^21,22^ Neither trial reported any evidence suggesting that weaning at 3-4 months compared with 6 months affected infant weight or length. Both trials had a sample size of <150 infants and were unable to control for the amount of complementary food offered to the participants.

The 15 observational studies that examined age at weaning between age 3 and 6+ months reported a variety of significant and nonsignificant associations. Ten of the 15 studies reported no association between age at weaning and at least 1 growth outcome.^13,15,17,23-29^ In addition, 10 of the 15 studies reported a significant inverse association between age at weaning and at least 1 growth outcome^12-14,16,17,27-31^; however, of these latter 10 studies, 4 did not consider the possibility of reverse causality by accounting for preweaning measurements.^12,13,17,31^ Of the remaining 6 studies, 1 found significant inverse associations with weight only before age 3 months,^27^ and a second study found an inverse association with weight gain in only 1 of several periods tested and only in breast-fed infants.^28^ The latter study furthermore used a group with very late age at weaning for comparison (4-6.3 months vs >6.5 months).^28^ A third study also compared very extreme weaning groups (3 months vs >6 months).^29^ The remaining 3 studies^14,16,30^ compared infants weaned at ≥4 months and those weaned at <4 months; the group of infants weaned at <4 months includes some with very early age at weaning.

Four studies reported evidence in support of reverse causality (ie, larger size or faster growth preceded earlier age at weaning). Van Rossem et al^15^ found a significant preweaning increase in weight-for-height in infants weaned between 3 and 6 months compared with infants weaned at 6 months or later, but no difference in weight-for-height at age 12 months. Baird et al^13^ found an inverse association between age at weaning and both weight and length at age 6 months; however, when accounting for preweaning weight and length gain, these associations were nonsignificant. Lande et al^26^ found an inverse association between age at weaning and ponderal index at birth, but no association with BMI at age 12 months. Finally, Wright et al^27^ reported inverse associations between age at weaning and weight up to 3 months, but no association with weight gain between 1.5 and 12 months.

The 2 observational studies that examined infants weaned at very early ages also provided evidence of reverse causality. Morgan et al^32^ found that infants weaned at 3 months were already heavier and longer than infants not yet weaned; this difference was no longer apparent at age 18 months. Similarly, Forsyth et al^33^ found that age at weaning was inversely associated with weight as early as 1 month, but found no association with weight at 13 or 26 months.

Three studies involving tests for interaction between age at weaning and type of milk feeding reported varying results. Baker et al^16^ reported a significant interaction between age at weaning and breastfeeding duration on weight gain between 0 and 12 months; longer duration of breastfeeding weakened the association between age at weaning and weight gain. Haschke and van't Hof^17^ also reported a significant interaction between age at weaning and breastfeeding duration, but only on weight and length gain between 1 and 4 months and not thereafter (the direction was not reported). Conversely, in a stratified analysis, Heinig et al^28^ found an inverse association between age at weaning and weight gain between 6 and 9 months in breastfed infants, but no association in formula-fed infants. Finally, 1 study reported nonlinear associations between age at weaning and infant growth,^23^ and another study reported a positive association between age at weaning and growth.^17^

## Discussion

Our primary analysis of a UK birth cohort study found significant inverse associations between age at weaning and weight and BMI at birth and age 3 months, before weaning had occurred. Therefore, age at weaning likely was influenced by mothers' responses to infant size, growth, or hunger cues. This finding is consistent with the most frequently reported reason for weaning in the UK Infant Feeding Survey: “Baby no longer satisfied with milk feeds.”^9^

Conversely, we found no evidence that earlier age at weaning, between 3 and 6 months, promotes subsequent infant weight gain or growth. Associations between age at weaning and both weight and length at 12 months became nonsignificant after adjusting for type of milk feeding and anthropometry before weaning. In addition, infants who exhibited faster weight gain between birth and 3 months were weaned earlier than those with average or slower weight gain. Type of milk feeding before weaning likely affects both time of weaning and infant growth.

The significant inverse associations between age at weaning and infant growth before weaning in our primary analysis are consistent with the existing literature.^15,17,26,27,32,33^ In addition, the pattern in our primary analysis of mixed significance after adjustment for only demographic confounders and attenuation on further consideration of confounding and reverse causality supports the null findings of the 2 randomized controlled trials.^21,22^ This pattern is also consistent with the significant inverse associations reported in 4 studies that did not consider reverse causality^12,13,17,31^ and in 6 studies that reported evidence of reverse causality.^13,15,26,27,32,33^ It is clear that milk feeding should be treated as a confounder, as it is in the majority of the published observational studies; however, there remains inconsistency in the reported interactions, or lack thereof, between age at weaning and type of milk feeding on infant growth.^16,17,28^

Strengths of our study include the timely assessment of growth and type of milk feeding at age 3 months, just before the range of age at weaning considered here. In addition, growth was accurately assessed at research clinics. A limitation is the relatively low level of deprivation compared with the national UK level; however, our study population was representative of its South Cambridgeshire setting. Ethnic diversity was also low. Finally, only approximately one-half of eligible mothers were included in this analysis. Nevertheless, the distribution of age at weaning, as well as the associations between age at weaning and various maternal and demographic factors, are similar to those reported in the 2010 UK Infant Feeding Survey.^9^

Based on our primary analysis and systematic review of both experimental and observational evidence, weaning between age 3 and 6 months appears to have neutral effects on infant growth in high-income countries. Earlier introduction of solids in infants of larger size or faster growth likely explains the apparent inverse associations between age at weaning and infant growth. Thus, the question regarding the optimal age at weaning between 3 and 6 months in high-income countries should be informed by other infant and maternal health outcomes.

## Figures and Tables

**Figure:**
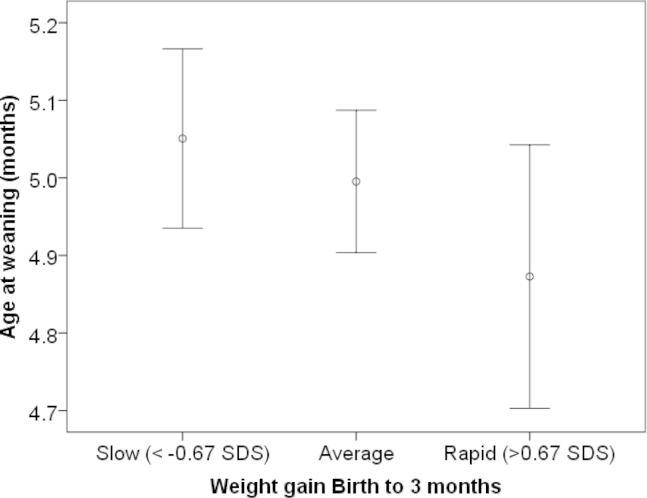
Age at weaning (mean ± 95% CI) by rate of weight gain between birth and 3 months. *P*_trend_ = .01, adjusted for age, sex, maternal age, parity, deprivation score, and birth weight. Infants weaned before age 3 months were excluded. Data are from the CBGS, 2001-2009.

**Table IV:** Associations between age at weaning and growth at birth and at 3 and 12 months, stratified by type of milk feeding at age 3 months

Variables	Model 1∗		Model 2†	
	B (95% CI)	*P* value	B (95% CI)	*P* value
Exclusively breast fed (n = 263)				
Weight *z*-score				
Birth	0.09 (−0.05 to 0.23)	.20	NA	
3 mo	0.07 (−0.06 to 0.21)	.29	0.03 (−0.09 to 0.16)	.59
12 mo	0.09 (−0.05 to 0.22)	.19	0.04 (−0.06 to 0.14)	.43
Length *z*-score				
Birth	−0.03 (−0.16 to 0.10)	.68	NA	
3 mo	0.05 (−0.11 to 0.20)	.54	0.07 (−0.05 to 0.19)	.27
12 mo	0.14 (−0.01 to 0.29)	.08	0.10 (0.00 to 0.21)	.05
BMI *z*-score				
Birth	0.14 (−0.04 to 0.33)	.12	NA	
3 mo	0.06 (−0.06 to 0.19)	.32	0.05 (−0.08 to 0.17)	.48
12 mo	0.02 (−0.11 to 0.14)	.81	−0.02 (−0.12 to 0.09)	.75
Exclusively or partially formula fed (n = 295)				
Weight *z*-score				
Birth	0.07 (−0.04 to 0.19)	.20	NA	
3 mo	0.11 (−0.01 to 0.23)	.06	0.07 (−0.03 to 0.17)	.16
12 mo	0.05 (−0.07 to 0.16)	.46	−0.04 (−0.12 to 0.05)	.39
Length *z*-score				
Birth	0.01 (−0.10 to 0.12)	.88	NA	
3 mo	0.06 (−0.07 to 0.19)	.39	0.05 (−0.06 to 0.16)	.35
12 mo	0.03 (−0.09 to 0.16)	.59	−0.01 (−0.09 to 0.08)	.90
BMI *z*-score				
Birth	0.13 (−0.01 to 0.28)	.07	NA	
3 mo	0.10 (−0.01 to 0.22)	.07	0.08 (−0.03 to 0.19)	.18
12 mo	0.03 (−0.08 to 0.14)	.57	−0.03 (−0.12 to 0.06)	.52

*NA*, not applicable. B, unstandardized regression coefficient; represents the change in *z*-score for each 1 month earlier weaning between 6 and 3 months.

∗ Adjusted for age, sex, maternal age, parity, and deprivation score.

† Model 1 with additional adjustment for the same growth measurement at the preceding time point.

**Table I:** Infant and maternal characteristics by age at weaning, CBGS, 2001-2009

Characteristics	Total no.	Age at weaning, mo				*P* value
		3.0-3.9	4.0-4.9	5.0-5.9	6.0-6.9	
Infant characteristics						
Sex, n (%)	571					<.01∗
Male	295	32 (72.7)	83 (56.8)	112 (49.6)	68 (43.9)	
Female	276	12 (27.3)	63 (43.2)	114 (50.4)	87 (56.1)	
Ethnicity, n (%)	500					.55†
White	472	33 (94.3)	113 (91.9)	193 (95.1)	133 (95.7)	
Not white	28	2 (5.7)	10 (8.1)	10 (4.9)	6 (4.3)	
Milk feeding at 3 mo, n (%)	558					<.01∗
Breast milk only	263	12 (29.3)	46 (32.9)	119 (53.6)	86 (55.5)	
Formula milk only	149	17 (41.5)	56 (40.0)	42 (18.9)	34 (21.9)	
Mixed	146	12 (29.3)	38 (27.1)	61 (27.5)	35 (22.6)	
Maternal characteristics						
Age, y, mean (SD)	569	33.5 (4.2)	32.6 (4.1)	33.7 (3.9)	34.3 (4.2)	<.01∗
Deprivation score, mean (SD)	569	9.4 (2.8)	9.5 (3.4)	8.9 (3.7)	8.9 (3.2)	.31
Smoking status, n (%)	570					.47†
Yes	8	1 (2.3)	3 (2.1)	1 (0.4)	3 (1.9)	
No	562	43 (97.7)	143 (97.9)	225 (99.6)	151 (98.1)	
Parity, n (%)	570					.19
1	254	18 (40.9)	62 (42.5)	113 (50.0)	61 (39.6)	
>1	316	26 (59.1)	84 (57.5)	113 (50.0)	93 (60.4)	
Marital status, n (%)	537					.24†
Married	458	33 (80.5)	107 (80.5)	193 (89.8)	125 (84.5)	
Single	7	1 (2.4)	2 (1.5)	3 (1.4)	1 (0.7)	
Cohabiting	72	7 (17.1)	24 (18.0)	19 (8.8)	22 (14.9)	
Education level, n (%)	233					.03†^,^‡
O-level	29	4 (16.0)	12 (21.4)	6 (6.5)	7 (11.9)	
A-level	44	5 (20.0)	13 (23.2)	21 (22.6)	5 (8.5)	
Degree+	160	16 (64.0)	31 (55.4)	66 (71.0)	47 (79.7)	
Prepregnancy BMI, n (%)	488					<.01∗^,^†
Underweight	14	0 (0.0)	1 (0.8)	7 (3.5)	6 (4.6)	
Normal	317	21 (53.8)	75 (63.0)	140 (70.4)	81 (61.8)	
Overweight	116	10 (25.6)	36 (30.3)	44 (22.1)	26 (19.8)	
Obese	41	8 (20.5)	7 (5.9)	8 (4.0)	18 (13.7)	

Percentages might not add up to exactly 100% because of rounding.

∗ *P* < .01.

† Pearson χ^2^ assumption that cells have an expected count >5 violated.

‡ *P* < .05.

**Table II:** Mean (SD) weight, length, and BMI *z*-scores at birth, 3 month, and 12 months by age at weaning, CBGS, 2001-2009

Variables	Age at weaning, mo				*P* value
	3.0-3.9	4.0-4.9	5.0-5.9	6.0-6.9	
Weight *z*-score					
Birth	0.17 (0.90)	0.23 (0.88)	−0.07 (0.96)	0.04 (0.93)	.02∗
3 mo	0.08 (0.92)	−0.03 (0.94)	−0.29 (0.94)	−0.30 (0.92)	.01∗
12 mo	0.59 (0.98)	0.58 (0.99)	0.39 (0.95)	0.25 (0.92)	.01∗
Length *z*-score					
Birth	−0.09 (0.88)	0.03 (0.89)	−0.18 (0.89)	0.00 (0.89)	.12
3 mo	0.09 (1.00)	0.18 (1.03)	−0.05 (1.02)	−0.08 (1.11)	.11
12 mo	0.26 (1.06)	0.48 (1.05)	0.23 (1.04)	0.00 (1.04)	<.01†
BMI *z*-score					
Birth	0.24 (1.15)	0.27 (1.11)	−0.06 (1.27)	0.01 (1.23)	.06
3 mo	0.04 (0.86)	−0.18 (0.93)	−0.36 (0.87)	−0.34 (0.85)	.02∗
12 mo	0.60 (0.93)	0.42 (0.94)	0.36 (0.83)	0.34 (0.84)	.33

∗ *P* < .05.

† *P* < .01.

**Table III:** Association between age at weaning and growth at birth, 3 months, and 12 months, CBGS, 2001-2009

Variables	Model 1∗		Model 2†		Model 3‡	
	B (95% CI)	*P* value	B (95% CI)	*P* value	B (95% CI)	*P* value
Weight *z*-score						
Birth	0.08 (0.00 to 0.17)	.05	NA		NA	
3 mo	0.12 (0.04 to 0.21)	.01§	0.08 (−0.01 to 0.17)	.07	0.04 (−0.03 to 0.12)	.27
12 mo	0.13 (0.04 to 0.21)	.01§	0.06 (−0.03 to 0.15)	.17	0.01 (−0.06 to 0.07)	.88
Length *z*-score						
Birth	0.00 (−0.08 to 0.08)	.98	NA		NA	
3 mo	0.08 (−0.02 to 0.17)	.12	0.05 (−0.05 to 0.14)	.37	0.05 (−0.03 to 0.13)	.24
12 mo	0.14 (0.05 to 0.24)	<.01¶	0.08 (−0.02 to 0.17)	.13	0.04 (−0.02 to 0.11)	.20
BMI *z*-score						
Birth	0.13 (0.02 to 0.24)	.02§	NA		NA	
3 mo	0.11 (0.03 to 0.19)	.01§	0.07 (−0.01 to 0.16)	.08	0.05 (−0.03 to 0.13)	.21
12 mo	0.06 (−0.02 to 0.14)	.13	0.02 (−0.06 to 0.11)	.56	−0.02 (−0.08 to 0.05)	.64

*NA*, not applicable. B, unstandardized regression coefficient; represents the change in *z*-score for each 1 month earlier weaning between 6 and 3 months.

∗ Adjusted for age, sex, maternal age, parity, and deprivation score.

† Model 1 with additional adjustment for milk feeding at 3 months.

‡ Model 2 with additional adjustment for the same growth measurement at the preceding time point.

§ *P* < .05.

¶ *P* < .01.

**Table V:** Summary of studies that examined effects of age at weaning on infant growth

Authors	Year	Sample size, country	Weaning age, mo	Reported effect or association with age at weaning	Potential Confounders Considered
Randomized controlled trials					
Jonsdottir et al^21^	2012	N = 100, Iceland	4 vs 6	No effect on weight gain from 0-4 (*P* = .51), 4-6 (*P* = .90), or 0-6 mo (*P* = .71) No effect on length gain from 0-4 (*P* = .40), 4-6 (*P* = .96), or 0-6 mo (*P* = .85)	NA
Mehta et al^22^	1998	N = 147, US	3-4 vs 6	No effect on weight at 3 (*P* = .47), 6 (*P* = .71), or 12 mo (*P* = .60) No effect on length at 3 (*P* = .50), 6 (*P* = .39), or 12 mo (*P* = .90)	NA
Observational studies					
Moss and Yeaton^12^	2014	N = 7200, US	<4, 4-5, ≥6	Inverse association. Compared with <4 mo, age at weaning 4-5 or ≥6 was associated with lower odds of BMI ≥95th percentile at 24 mo (*P* < .001 and *P* < .05)	Sex, BW, poverty, race, maternal education, maternal age, BF
van Rossem et al^15^	2013	N = 3184, The Netherlands	0-3, 3-6, ≥6	No association with change in weight-for-height after weaning to 12 mo Other: Before weaning, infants weaned at 3-6 mo had a larger increase in weight-for-height (*P* < .05) than those weaned at 0-3 or ≥6 mo.	Age, sex, maternal education, ethnicity, maternal BMI, smoking in pregnancy, BF, food allergies, hospitalizations
Grote et al^23^	2011	N = 671, Europe	≤3.25, 3.5-4.25, 4.5-5.25, >5.5	Nonlinear association with weight (*P* = .027) and length (*P* = .049) at 24 mo Nonlinear association with weight-for-age (*P* = .005) and BMI-for-age (*P* = .011) trajectories at 0-24 mo No association with BMI (*P* = .220) or weight-for-length (*P* = .127) at 24 mo No association with weight-for-length (*P* = .084) or length-for-age (*P* = .127) trajectories at 0-24 mo	Age, sex, size at ∼2 weeks, and country of birth Only formula fed infants
Mihrshahi et al^24^	2011	N = 612, Australia	<4, ≥4	No association with rapid weight gain between birth to 4-7 mo (*P* = .476)	Age, sex, maternal BMI, and maternal age
Baird et al^13^	2008	N = 1733, UK	<3, 3, 4, ≥5	Inverse association with weight (*P* = .008) and length (*P* = .002) at 6 mo No association with weight gain (*P* = .345) or length gain (*P* = .111) at 0-6 mo	Sex, maternal education, parity, smoking in pregnancy, BF
Sloan et al^14^	2008	N = 216, Ireland	<4, ≥4	Inverse association with weight at 7 mo (*P* = .046), weight at 14 mo (*P* = .035), and weight gain in 2-14 mo (*P* = .029) Other: no association with weight at birth (*P* = .15) or 2 mo (*P* = .56)	Age, sex, BF
Kupperberg and Evers^25^	2006	N = 102, Canada	Not categorized	No association with BMI >85th percentile at 3 or 18 mo (*P* > .05)	
Lande et al^26^	2005	N = 1441, Norway	≤4, 4-5, >5	No association with BMI at 12 mo Other: Inverse association with ponderal index at birth (*P* = .02)	Sex, size at birth, BF, diet at 12 mo.
Baker et al^16^	2004	N = 3768, Denmark	<4, ≥4	Inverse association with weight gain 0-12 mo (*P* = .0019) Other: No association with birth weight (*P* = .50); weaker effect with longer BF duration (interaction *P* = .0021)	Age, sex, parity, gestational weight gain, duration of gestation, smoking in pregnancy, BW, length at 1 yr
Morgan et al^32^^,^∗	2004	N = 1694, UK	≤3, >3	Positive association with weight gain (*P* = .020) and length gain (*P* = .011) at 3-18 mo Inverse association with weight (*P* < .001) and length (*P* = .01) at 3 mo. No association with weight or length at 18 mo	Sex, size at 3 mo, size for gestational age at birth, BF
Wright et al^27^	2004	N = 707, UK	Not categorized	Inverse association with weight gain 0-1.5 mo (*P* < .001) and weight at 0 (*P* = .04), 1.5 (*P* < .001), and 3 mo (*P* < .001) No association with weight gain at 1.5-12 mo (*P* = .45)	
Haschke and van't Hof^17^	2000	N = 504, Europe	<4, ≥4	Positive association with length gain from 1-24 mo (*P* = .01), weight gain from 1-12 mo (*P* = .002), BMI gain from 1-12 mo (*P* = .002), BMI at 1-18 mo (*P* < .05), and weight at 3-6 mo (*P* < .05) Inverse association with length at 1-3 and 5-6 mo (*P* < .05) No association with length gain at 1-12 mo, weight gain at 1-24 mo, or BMI gain at 1-24 mo Other: Significant interaction between age at weaning and breastfeeding duration only from 1-4 mo	Age, sex, maternal education, mid-parental height, BF
Forsyth et al^33^^,^∗	1993	N = 671, Scotland	<2, 2-3, >3	Inverse association with weight at 1 (*P* = .001), 2 (*P* = .003), 3.25 (*P* = .006), and 6.5 mo (*P* = .009) No association with weight at 13 mo (*P* = .30) or 26 mo (*P* = .70)	Sex, BW, maternal height, BF
Heinig et al^28^	1993	N = 105, US	4-6.5, ≥6.5	Inverse association with weight gain at 6-9 mo (*P* < .05) in BF infants No association with weight gain at 0-4, 4-6, or 9-12 mo or length gain at 0-12 mo in BF infants No association with weight or length gain in formula-fed infants	Sex, weight gain from 0-4 mo. Stratified by BF
Whitehead et al^30^	1986	N = 37, UK	<4, ≥4 (boys) <5, ≥5 (girls)	Inverse association with weight and length trajectories at 6-12 mo	Age, sex Only BF infants
Kramer et al^31^	1985	N = 361, Canada	2, 4, 6	Inverse association with weight at 6 mo (*P* < .001) and 12 mo (*P* < .001)	Age, sex, BW
Salmenpera et al^29^	1985	N = 198, Finland	3, >6	Inverse association with length gain 3-6 mo (*P* < .01), 6-9 mo (*P* < .05), and 9-12 mo (*P* < .001), and weight gain at 6-9 mo (*P* < .01) and 9-12 mo (*P* < .001) No association with length gain at 0-3 mo, weight gain at 0-6 mo, length at 9 mo, or weight at 9 mo	

*BW*, birth weight; *BF*, breast-feeding.

∗ Studies examined association only with very early weaning.
